# The “yeast-drop” treatment protocol in the *Drosophila* model for rapid and cost-effective TB drug testing

**DOI:** 10.3389/ftubr.2026.1746664

**Published:** 2026-01-27

**Authors:** Maria Vidal, Esther Fuentes, Nerea Escobar, Marta Arch, Pere-Joan Cardona

**Affiliations:** 1 Tuberculosis Research Unit, Germans Trias i Pujol Research Institute (IGTP), Badalona, Catalonia, Spain; 2 Department of Genetics and Microbiology, Universitat Autònoma de Barcelona, Bellaterra, Catalonia, Spain; 3 Comparative Medicine and Bioimage Centre of Catalonia (CMCiB), Germans Trias i Pujol Research Institute (IGTP), Badalona, Catalonia, Spain; 4 Laboratori Clínic Metropolitana Nord, Microbiology Department, Germans Trias i Pujol University Hospital (HUGTP), Badalona, Spain; 5 Centro de Investigación Biomédica en Red en Enfermedades Respiratorias (CIBERES), Instituto de Salud Carlos III (ISCIII), Madrid, Spain

**Keywords:** *Drosophila melanogaster*, methodology, mycobacteria, treatment, tuberculosis

## Abstract

**Background:**

Pulmonary tuberculosis (TB), caused by *Mycobacterium tuberculosis*, is the leading infectious disease globally. The lengthy treatment regimen and the potential side effects increase the probability of relapse and of developing drug resistance. These factors highlight the need for new therapeutic strategies, including host-directed therapies and anti-virulence approaches. However, the drug discovery pipeline is often limited by the simplicity of *in vitro* models and the cost and scalability challenges of mammalian *in vivo* models. In this study, we developed a cost-effective administration method using the *Drosophila melanogaster*–*Mycobacterium marinum* infection model called “yeast-drop”. This approach facilitates oral delivery and reduces the quantity of compound currently needed for treatment in the fly model.

**Methods:**

We compared the yeast-drop methodology with the standard method commonly used in *Drosophila* studies. Additionally, we assessed the efficacy of benchmarking antibiotics, host-directed therapies (HDTs), and anti-virulence compounds for TB treatment by measuring fly survival and bacterial burden.

**Results:**

Flies treated with the “yeast-drop” method showed a significant improvement in survival probability and a reduction in colony-forming units (CFUs) compared to non-treated flies. This was comparable to the results achieved with the standard feeding method. Among the compounds tested, linezolid proved to be the most effective antibiotic. HDTs such as aspirin, metformin, and simvastatin also enhanced survival rates and reduced CFUs following treatment, demonstrating conserved immune and metabolic mechanisms between flies and mammals. Similarly, BBH7 and ethoxzolamide, which act as anti-virulence agents, further reinforce the translational value of this type of treatment in the *Drosophila* model.

**Conclusion:**

Overall, the *Drosophila*–*M. marinum* model, combined with the yeast-drop methodology, offers a reliable, low-cost, and biologically relevant platform for early-stage screening of antimycobacterial, host-directed, and anti-virulence compounds, effectively bridging the gap between *in vitro* systems and mammalian models.

## 1 Introduction

Pulmonary tuberculosis (TB), caused by *Mycobacterium tuberculosis*, remains the leading infectious disease worldwide, with over 10 million new infections annually and approximately 1.25 million deaths (1). Although curable, the current treatment regimen presents major challenges. Drug-susceptible TB is treated with a 6-month regimen consisting of isoniazid, rifampicin, ethambutol, and pyrazinamide for the first 2 months, followed by isoniazid and rifampicin administration for an additional 4 months (1, 2). Drug-resistant TB requires 9–20-month regimens including newer or second-line agents such as bedaquiline, pretomanid, linezolid, and fluoroquinolones (1, 2). These lengthy treatments increase adverse effects and rates of non-adherence, leading to treatment failure, relapse, and the emergence of multidrug-resistant strains, which now account for hundreds of thousands of cases globally each year (1, 3). This situation underscores the urgent need for new therapeutic strategies beyond conventional antimicrobials.

The immune system plays a crucial role in controlling mycobacterial infections, and immunomodulatory strategies have shown promise in preclinical studies. In recent years, therapeutic research in TB has expanded beyond direct antimicrobial agents to include host-directed therapies (HDTs), which aim to modulate the host immune response to enhance pathogen clearance and reduce tissue damage, which may enhance treatment efficacy, shorten therapy duration, and lower the risk of resistance emergence (4–6). Despite their potential, HDTs have yet to be implemented in routine clinical management of TB, highlighting a gap between research advances and therapeutic application (7, 8). In parallel, anti-virulence strategies are also gaining traction as complementary approaches that aim to disarm the pathogen rather than kill it, potentially reducing selective pressure for resistance (9, 10).

The development of these innovative strategies relies on robust and scalable screening platforms. *Drosophila melanogaster* has emerged as a powerful model organism for studying host–pathogen interactions and assessing therapeutic efficacy. Its short life cycle, low maintenance cost, genetic tractability, and conserved innate immune mechanisms make it an attractive intermediary model between *in vitro* assays and mammalian models (11, 12). In the context of mycobacterial infections, *Mycobacterium marinum* and *Mycobacterium abscessus* infections in *Drosophila* have been successfully employed to validate the *in vivo* efficacy of multiple compounds (13–16). Despite its potential, the *Drosophila* model remains underutilized in therapeutic pipelines, often excluded from standard screening cascades. One of the key limitations hindering its broader adoption is the large amount of compound required to achieve effective concentrations in fly feeding media, which limits large-scale screening mainly due to high production costs (12, 17, 18).

In a previous methodological review conducted by our group, we summarized existing treatment pipelines using *D. melanogaster* infection model. We identified yeast as a rewarding substrate commonly used in *Drosophila* husbandry (e.g., for rearing embryos or larvae), but it had not been previously applied in treatment screenings (19, 20). Building on this observation, we explored whether yeast could be used as a delivery vehicle to overcome the aforementioned limitation. To our knowledge, this is the first time yeast has been implemented as a rewarding substrate for compound delivery in treatment experiments.

In this manuscript, we describe and validate the yeast-based compound delivery method by testing benchmark compounds currently used for tuberculosis treatment in a *M. marinum*-infected *Drosophila* model. Our results demonstrated the potential of this approach to enhance the utility of *Drosophila* as a practical and cost-effective *in vivo* screening platform. This platform can be integrated into drug discovery pipelines for antimicrobial, host-directed, and anti-virulence therapies, while also incorporating sex-stratified analyses to capture biologically relevant differences in treatment response.

## 2 Materials and methods

### 2.1 Mycobacterial strain and culture conditions

We did all the experiments with a *Mycobacterium marinum* E11 strain resistant to kanamycin (a kind gift from Wilbert Bitter, Vrije Universiteit Amsterdam) (21). The mycobacterial strain was cultured in 7H9 complete medium supplemented with 20 μg/ml of kanamycin and incubated at 30 °C with constant agitation (150 rpm) for 10 days, until an optical density at 600 nm of 1.5 was reached. The cultures were then centrifuged for 5 min at 5,000g, resuspended in phosphate-buffered saline (PBS) with 0.2% Tween 80, and centrifuged again for 5 min at 500g to remove clumps. Supernatants were transferred to a new tube, centrifuged for 5 min at 5,000g, and resuspended in 1ml of 7H9 with 15% glycerol. Mycobacterial cultures were then aliquoted and frozen at −80 °C. Each stock was titrated after being frozen for at least one night. For titration, we performed serial dilutions of the frozen stocks and cultured them in 7H10 media supplemented with 20 μg/ml of kanamycin and 1.25 μg/ml of Amphotericin B.

### 2.2 *Drosophila* stock and maintenance

*Drosophila melanogaster* Oregon-R-C wild-type flies were obtained from the Bloomington Drosophila Stock Center (Indiana University). Flies were raised on a standard cornmeal medium (Nutri-Fly^®^ Bloomington Formulation, Genesee Scientific) prepared following the manufacturer's instructions and maintained at 24 ± 1 °C, 65–70% humidity with a 12 h light/dark cycle. Male and female flies were aged for 3–5 days post-eclosion before experimentation. For all *in vivo* manipulation, flies were anesthetized with CO_2_.

### 2.3 Systemic infection of adult *Drosophila*

To prepare for systemic infections, an aliquot *M. marinum* E11 was defrosted and centrifuged at 5,000g for 5 min. The resulting pellet was rinsed with sterile PBS and diluted to achieve an inoculation dose of 50 Colony Forming Units (CFUs) per fly. The final inoculum contained 25 μl of brilliant blue dye to visually ensure its introduction into the fly's body. We injected 13.8 nl of the inoculum solution into the abdomen of anesthetized flies using a nano-injector (Nanoject II, Drummond). For survival analyses, deaths occurring within the first 48 h post-injection were attributed to procedure-related wounding and were excluded (22).

### 2.4 Treatment preparation

**Figure 1:**
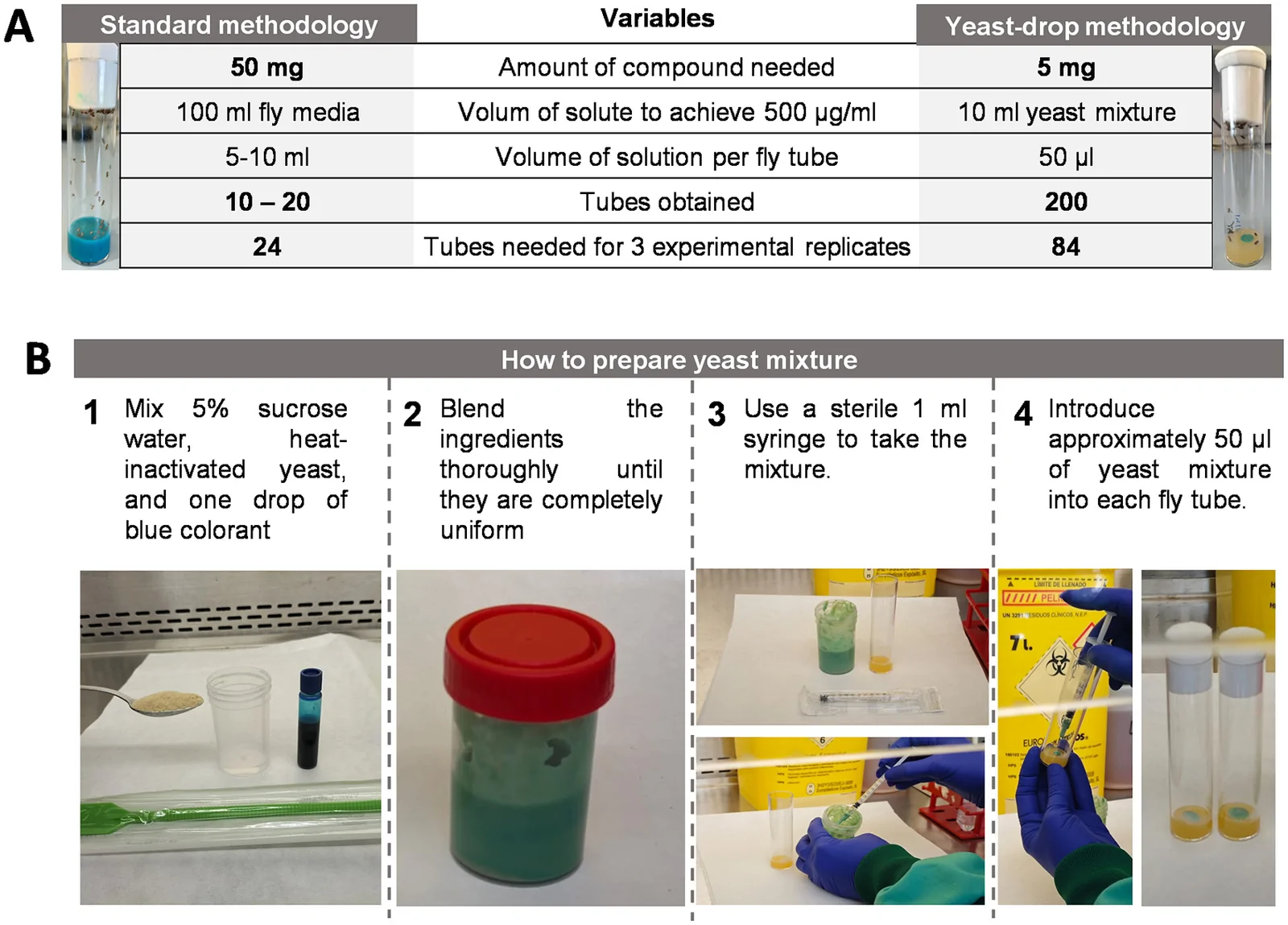
Overview of the yeast-drop methodology for treating *Drosophila melanogaster*. **(A)** Table summarizing the variables that differ between the two treatment methodologies: the standard method **(left)** and the yeast-drop method **(right)**, along with their numerical specifications. **(B)** Step-by-step illustration of the preparation of the yeast mixture used in the yeast-drop methodology.

To prevent significant compound losses from directly mixing into the fly medium, we developed a more cost-effective treatment protocol, referred to in the manuscript as the “yeast-drop” protocol. It involved creating a mixture of yeast, water with 5% sucrose, and the appropriate concentrations of each compound. We mixed these ingredients until a uniform semi-solid solution was formed. Next, we used a syringe to place 50 μl of this mixture in the center of a *Drosophila* tube filled with minimal media. To prepare 1 l of minimal media, we mixed 500 ml of juice with 500 ml of distilled water. We then added 60 g of glucose and 30 g of bacteriological agar. After bringing it to a boil and mixing thoroughly, we added 1.5 ml of propionic acid. Finally, we filled *Drosophila* tubes with the prepared solution. We utilized 35 ml *Drosophila* tubes for the treatment experiments, further enhancing the cost-effectiveness of the protocol (Figure 1).

The treatment tubes for the standard methodology used in the study were prepared as follows. We prepared a standard cornmeal medium using the Nutri-Fly^®^ Bloomington Formulation from Genesee Scientific, following the manufacturer's instructions. After autoclaving the medium, we allowed it to cool before adding 1.5 ml of propionic acid and sucrose (to achieve a final concentration of 5%), along with linezolid or rifampicin to reach a concentration of 500 μg/ml. Figure 1A shows the amount of compound required for each treatment method. Using the yeast-drop method, we reduced the required amount tenfold (Figure 1A).

The benchmark compounds used in this study were formulated in powder and can be categorized into three groups based on their mode of action. The antibiotics tested included isoniazid, moxifloxacin, pyrazinamide, rifampicin, and linezolid, each administered at a concentration of 500 μg/ml. In addition, the Standard of Care combination HRZE was tested at a total concentration of 500 μg/ml using the commercially available formulation Rimstar^®^, a fixed-dose combination containing rifampicin (150 mg), isoniazid (75 mg), pyrazinamide (400 mg), and ethambutol hydrochloride (275 mg) per tablet. For HDTs, aspirin and ibuprofen were used at 1 μM, while doramapimod, simvastatin, and metformin were tested at 10 μM. Lastly, the anti-virulence compounds, ethoxzolamide (a PhoP inhibitor) and BBH7 (an ESX-1 inhibitor), were both applied at a concentration of 10 μM. No dose-response assays were performed for dose selection in this study. Instead, concentrations were chosen based on a prior literature review conducted by our group, using the doses previously reported as biologically active for each specific compound (12). As this study serves a validation purpose, any observed efficacy must be further confirmed through dose–response analyses.

### 2.5 Experimental design for treatment experiments

Flies were systematically infected with 50 CFUs of *M. marinum* as described above and placed into standard cornmeal tubes. On day 3 post-infection, when the infection by *M. marinum* in the fly was established, flies were transferred to smaller tubes containing minimal media along with 50 μl of yeast paste (“yeast-drop” methodology), with or without the tested compound. Flies infected with *M. marinum* and given yeast paste without compounds served as the control for the treatment experiments. A total of 120 individuals (60 males and 60 females) were assigned to each condition, with 30 males and 30 females used for survival analysis, and the other 30 males and 30 females used for CFU quantification. The flies were maintained in the treatment tubes for 7 days, with the tubes being changed and the treatment refreshed daily. We monitored survival daily and assessed bacillary load on day 10 post-infection, right after the treatment. The survival of the flies was tracked for a total of 25 days. For the set-point bacillary load, on the specified day, 5 males and 5 females per condition were individually collected, washed with 70% ethanol, and rinsed twice with PBS. Each fly was then mechanically homogenized into 200 μl of sterile PBS, diluted, and plated onto 7H10 plates supplemented with kanamycin (20 μg/ml) and Amphotericin B (1.25 μg/ml), and incubated for 10 days at 30 °C. All experiments were conducted in triplicate, except for the ibuprofen survival and CFU, as well as the BBH7 CFU, which were performed in duplicate. The same protocol was applied to those experiments with standard treatment tubes, with the difference that these were changed every 3 days, and not daily. As part of the validation of the new methodology, toxicity studies were also conducted by administering linezolid and rifampicin at 500 μg/ml to non-infected flies for 7 days using both the standard and the yeast-drop methodologies, while monitoring survival throughout the exposure period. Toxicity experiments were performed in duplicate.

### 2.6 Statistical analysis

All statistical analyses were performed using R software (version 4.3.1; R Foundation for Statistical Computing, Vienna, Austria). Data visualization and graph generation were also carried out in R using dedicated packages for survival analysis and data plotting. Data for each condition were collected across independent experimental rounds performed in duplicate or triplicate, with each round including its own control group. The statistical unit in all the analyses was the individual fly. Survival data from efficacy studies were analyzed using Cox proportional hazards regression models to estimate hazard ratios (HR) and their corresponding 95% confidence intervals (CIs). HRs were visualized using forest plots. To assess sex differences between groups, Estimated Marginal Means (EMMs) of log-HR were computed. Pairwise contrasts (female vs. male) were calculated within each treatment group. In toxicity studies, survival data were evaluated using Kaplan–Meier survival curves, and differences between groups were assessed with the log-rank (Mantel–Cox) test. CFU quantification data were analyzed using the non-parametric Kruskal–Wallis test, followed by *post hoc* pairwise multiple comparisons with Bonferroni correction for multiple testing. All statistical tests were two-tailed, and a *p*-value <0.05 was considered statistically significant. The technical limit of detection (LOD) for CFU experiments was 40 CFU/fly. CFU values below this threshold were considered below the LOD and were not interpreted as measured zero values. For graphical representation, log10 transformation, and statistical analysis, values below the LOD were imputed at the LOD value (40 CFU per fly), and are indicated as such in the figures. All analyses were performed grouped by compound category (antibiotics, HDTs, and anti-virulence) and by sex. Because experimental rounds were not organized by compound category and not all treatments were tested at the same time, control groups from different experimental rounds were pooled for graphical representation and statistical analysis. The consistency of results across experimental rounds was assessed by analyzing each round separately, as shown in the Supplementary Figure 1.

## 3 Results

### 3.1 The “yeast-drop” methodology supports compound efficacy screening in *M. marinum*-infected *Drosophila* while revealing sex-specific responses to treatment conditions

The standard methodology currently used for treatment screening in *Drosophila* involves mixing compounds directly into the fly food medium. While effective, this approach is cost-prohibitive when high compound concentrations are required, particularly for antimicrobials. To overcome this limitation, we developed an alternative treatment method, referred in the study as “yeast-drop,” in which compounds are incorporated into a yeast paste, and 50 μl of this mixture is placed on minimal media. To assess the performance of the yeast-drop method, we conducted a comparative experiment using both the standard and yeast-drop approaches. Flies were infected with *M. marinum* and, 3 days post-infection, were treated with 500 μg/ml of linezolid or rifampicin using either methodology. Non-treated flies served as negative controls for both treatment methodologies.

**Figure 2:**
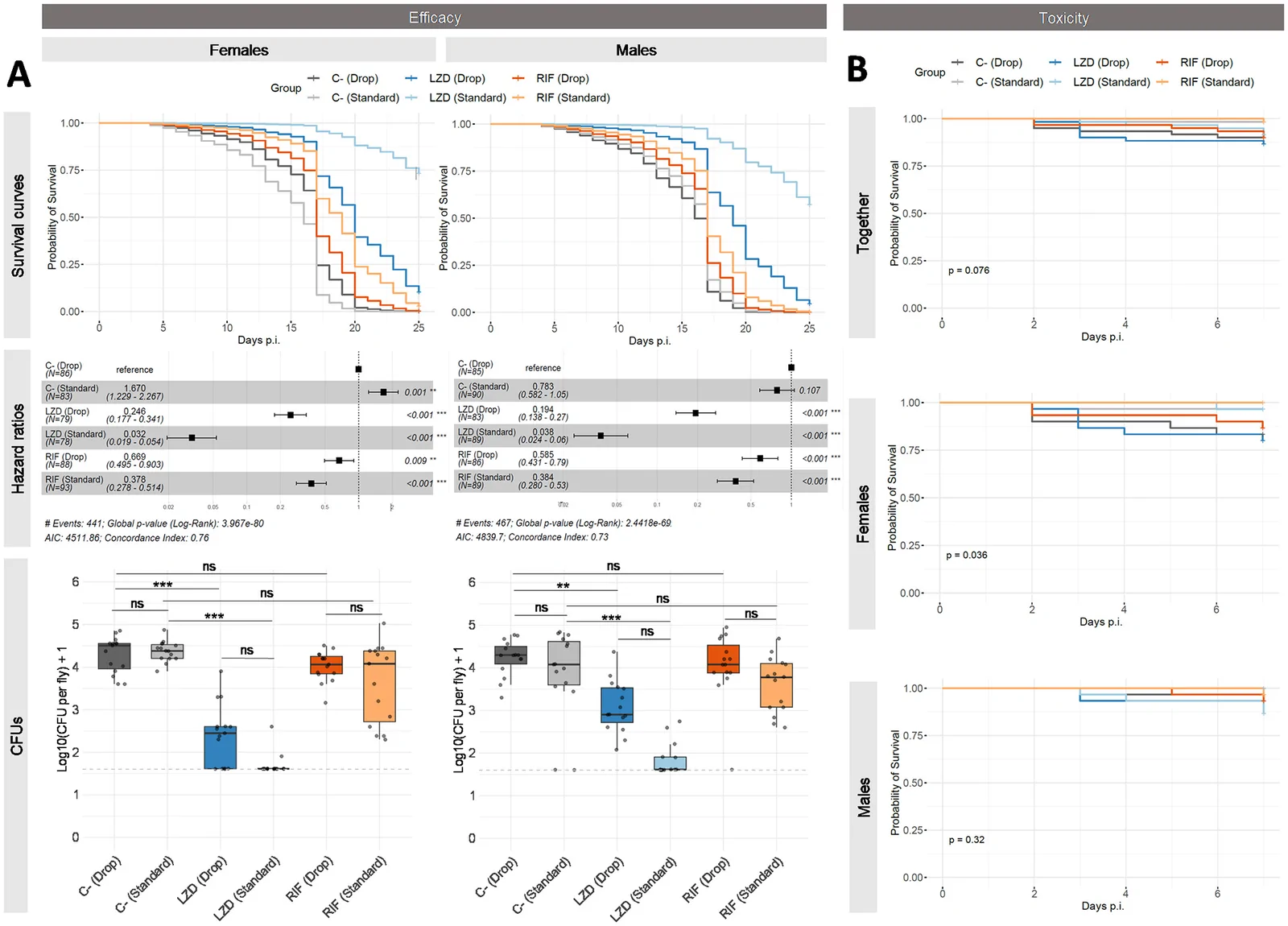
Validation of the “yeast-drop” treatment methodology in *M. marinum*-infected *Drosophila melanogaster*. **(A)** Efficacy studies. Probability of survival **(top)**, hazard ratios **(middle)**, and bacterial load expressed as log_10_ CFU per fly **(bottom)** in non-treated control flies (C–) and flies treated with 500 μg/ml linezolid (LZD) or rifampicin (RIF) using either the standard or yeast-drop (drop) methodologies. Analyses were performed separately for females **(left)** and males **(right)**. Survival data were analyzed using Cox proportional hazards regression, and CFU data using the Kruskal–Wallis test followed by Dunn's *post hoc* multiple comparisons with Bonferroni correction. The technical limit of detection (LOD) for CFU quantification is 40 CFU per fly and is indicated by a gray dotted line in all CFU plots. Values below this threshold are reported as “below LOD” and, where required for log_10_ transformation and statistical analysis, were imputed at the LOD value. **(B)** Assessment of toxicity related to methodology in non-infected flies. Kaplan–Meier survival curves showing overall survival **(top)**, and survival stratified by females **(middle)** and males **(bottom)** after 7 days of exposure to the standard or yeast-drop methodologies, with or without antibiotic treatment. Statistical differences between groups were assessed using the log-rank (Mantel–Cox) test. Statistical significance is indicated as follows: ns, not significant; *p < 0.05; **p < 0.01; ***p < 0.001.

Survival analysis demonstrated that both the standard and yeast-drop methodologies significantly increased survival probability following both linezolid and rifampicin treatment in both sexes compared with controls (hazard ratios and *p*-values are provided in Figure 2A). Quantification of bacillary load revealed a significant reduction after linezolid treatment using both methodologies in both sexes (yeast-drop: median 2.45 log_10_ CFU/fly, *p* < 0.001 for females; 2.90 log_10_ CFU/fly, *p* = 0.007 for males; standard: below LOD log_10_ CFU/fly, *p* < 0.001 for females; 1.61 log_10_ CFU/fly, *p* < 0.001 for males; Figure 2A) compared with their respective methodological controls. In contrast, rifampicin-treated flies showed bacillary loads that were not significantly different from controls under either methodology (Figure 2A). When comparing the efficacy of the two methodologies within each treatment group, the standard method demonstrated significantly reduced hazard ratios (HR = 0.16, 95% CI 0.12–0.22, *p* < 0.001 for LZD; HR = 0.61, 95% CI 0.49–0.75, *p* < 0.001 for RIF), demonstrating greater efficacy than the yeast-drop method, although this difference was not statistically significant in CFU counts (Figure 2A, Supplementary Figure 2). Interestingly, in females, infected but non-treated flies fed using the yeast-drop methodology survived significantly longer than those fed by the standard method (HR = 1.67, 95% CI 1.23–2.27, *p* = 0.01; Figure 2A).

In order to further explore the sexual dimorphism in our data, we also analyzed survival and CFU data separately by sex. Female flies consistently showed lower hazard ratios than males across treatment groups, indicating better survival outcomes for females when treated. This trend was consistent for both treatment methodologies (Yeast-drop: HR = 0.63, 95% CI 0.47–0.86, *p* = 0.003 for C–; HR = 0.73, 95% CI 0.54–1.00, *p* = 0.053 for LZD; HR = 0.68, 95% CI 0.51–0.93, *p* = 0.013 for RIF. Standard: HR = 1.38, 95% CI 1.02–1.86, *p* = 0.033 for C–; HR = 0.56, 95% CI 0.32–0.95, p = 0.033 for LZD; HR = 0.56, 95% CI 0.42–0.76, *p* < 0.001 for RIF; Supplementary Figure 3A). When comparing bacterial load, only those flies treated with linezolid using the yeast-drop methodology revealed lower median CFU counts in females compared with males (median = below LOD log_10_ CFU/fly; Dunn's *post hoc* test, Bonferroni correction, *p* = 0.21 for standard; 2.45 log_10_ CFU/fly, *p* = 0.01 for yeast-drop; Supplementary Figure 3B). These findings suggest a sex-dependent difference in infection control.

To determine whether the choice of treatment methodology influenced outcomes through toxicity effects, we conducted additional studies in non-infected flies. Flies were exposed to linezolid, rifampicin, or no compound for 7 days using both the standard and yeast-drop methodologies, and survival was monitored throughout the exposure period. Toxicity results showed that female flies exposed to the yeast-drop methodology exhibited a significant decrease in survival probability across both control and treated groups (log-rank = 11.9, d*f* = 5, *p* = 0.036). In contrast, male flies showed no significant differences among groups (log-rank = 5.9, d*f* = 5, *p* = 0.32; Figure 2B). These findings suggest that the yeast-drop methodology may elicit an adverse response in females, which may contribute to reduced survival in this group.

Together, these findings suggest that the yeast-drop methodology is an effective alternative for compound screening at higher doses. It consistently differentiates compound efficacy compared to non-treated controls while substantially reducing experimental costs compared to the standard method.

### 3.2 Benchmark therapeutic compounds against TB showed efficacy in a *M. marinum*-infected *Drosophila* model using the yeast-drop methodology

To further validate the yeast-drop methodology within the context of tuberculosis treatment, we benchmarked a range of compounds currently used or investigated for TB therapy, including antibiotics, HDTs, and anti-virulence compounds. The experimental procedure followed the same scheme described above. *M. marinum*-infected flies were treated 3 days post-infection with antibiotics: isoniazid, linezolid, moxifloxacin, pyrazinamide, rifampicin, and HRZE (representing the SoC for drug-susceptible TB). HDTs tested included aspirin, doramapimod, ibuprofen, metformin, and simvastatin, while anti-virulence compounds included BBH7 and ethoxzolamide, a PhoP inhibitor. Treatments were administered for 7 days, and CFU counts were quantified immediately after treatment. Survival monitoring was performed for 25 days. For clarity, results were presented grouped by compound category rather than by experimental round; therefore, controls were pooled as explained in the Methods section. Consistency across experimental rounds was verified by stratified analyses shown in the Supplementary Figure 1.

**Figure 3:**
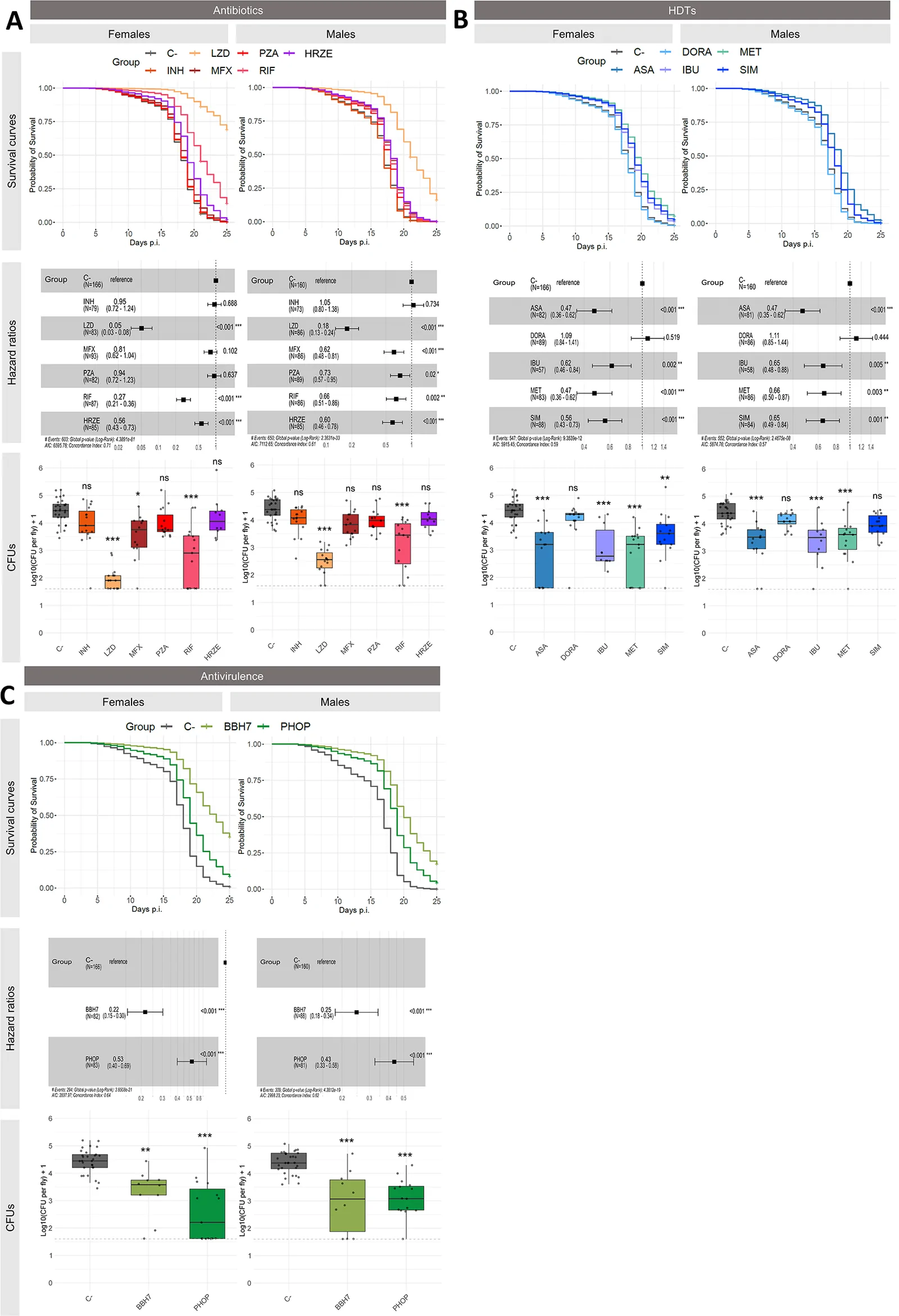
Efficacy of benchmarking compounds against TB using the yeast-drop treatment methodology in *M. marinum*-infected *D. melanogaster* model. Probability of survival **(top)**, hazard ratios **(middle)**, and bacterial load expressed as log_10_ CFU per fly **(bottom)** in non-treated control flies (C–) and flies treated with: **(A)** antibiotics-500 μg/ml isoniazid (INH), linezolid (LZD), moxifloxacin (MFX), pyrazinamide (PZA), rifampicin (RIF), and the current standard of care (HRZE); **(B)** Host-directed therapies (HDTs)-1 μM aspirin (ASA) and ibuprofen (IBU), and 10 μM doramapimod (DORA), metformin (MET), and simvastatin (SIM); **(C)** antivirulence compounds-10 μM ESX-1 inhibitor BBH7 and the PhoP inhibitor ethoxozolamide (PHOP). Analyses were performed separately for females **(left)** and males **(right)**. Survival data were analyzed using Cox proportional hazards regression, and CFU data using the Kruskal–Wallis test followed by Dunn's *post hoc* multiple comparisons with Bonferroni correction. The technical limit of detection (LOD) for CFU quantification is 40 CFU per fly and is indicated by a gray dotted line in all CFU plots. Values below this threshold are reported as “below LOD” and, where required for log_10_ transformation and statistical analysis, were imputed at the LOD value. Statistical significance is indicated as follows: ns, not significant; *p < 0.05; **p < 0.01; ***p < 0.001.

For antibiotics, the survival probability was significantly increased in most groups, except for isoniazid in males. In females, survival curves were significantly improved for linezolid, rifampicin, and HRZE, but not for isoniazid, moxifloxacin, or pyrazinamide. Across both sexes, linezolid was the most effective compound (HR = 0.051, 95% CI 0.033–0.077, *p* < 0.001 for females; HR = 0.18, 95% CI 0.13–0.24, *p* < 0.001 for males). Hazard ratios and *p*-values for all groups are provided in Figure 3A. Analysis of CFU counts revealed that linezolid (median = 1.90 log_10_ CFU/fly; Dunn's *post hoc* test, Bonferroni correction, *p* < 0.001), moxifloxacin (3.76 log_10_ CFU/fly, *p* = 0.02), and rifampicin (2.90 log_10_ CFU/fly, *p* < 0.001) significantly reduced bacterial burden in females when compared with controls. In contrast, in males, only linezolid (2.56 log_10_ CFU/fly, *p* < 0.001) and rifampicin (3.45 log_10_ CFU/fly, *p* < 0.001) produced significant reductions. Although the CFU counts for isoniazid, pyrazinamide, and HRZE were lower than the control median, these decreases were not statistically significant in either sex; moxifloxacin also failed to show a significant reduction in males (Figure 3A). The inability to detect efficacy for certain benchmark compounds can be attributed to physiological factors related to both the host and the bacteria. These findings demonstrated that the yeast-drop methodology effectively discriminates the efficacy of TB antibiotics when surpassing the physiological limitations of the model.

In the case of HDTs, survival probability was significantly increased after treatment of *Drosophila* infected with *M. marinum* using the yeast-drop methodology with aspirin, ibuprofen, metformin, and simvastatin in both males and females. No significant differences compared to the control group were observed in flies treated with doramapimod in either sex. Hazard ratios and *p*-values for all groups are provided in Figure 3B. Regarding bacillary load, doramapimod was the only compound that failed to significantly reduce CFU counts compared with controls in both sexes, while simvastatin showed no significant effect in males. Treatment with aspirin (3.2 log_10_ CFU/fly, *p* < 0.001 for females; 3.5 log_10_ CFU/fly, *p* < 0.001 for males), ibuprofen (2.79 log_10_ CFU/fly, *p* < 0.001; 3.51 log_10_ CFU/fly, *p* < 0.001), metformin (3.20 log_10_ CFU/fly, *p* < 0.001; 3.60 log_10_ CFU/fly, *p* < 0.001), and simvastatin in females (3.60 log_10_ CFU/fly, *p* = 0.003; 3.92 log_10_ CFU/fly, *p* = 0.21) significantly reduced bacterial burden after treatment compared to controls (Figure 3B). These findings demonstrate that HDTs can be effectively evaluated for efficacy in the *Drosophila* infection model, while also highlighting structural or pharmacological limitations—such as those associated with doramapimod, a p38 MAPK inhibitor—that may influence compound performance.

For anti-virulence compounds, both BBH7 and ethoxzolamide significantly improved survival in *Drosophila* females and males (hazard ratios and *p*-values are provided in Figure 3C). Both compounds also significantly reduced bacillary load compared with non-treated controls (BBH7: 3.58 log_10_ CFU/fly, *p* = 0.002 for females; 3.13 log_10_ CFU/fly, *p* < 0.001 for males; Ethoxzolamide: 2.20 log_10_ CFU/fly, *p* < 0.001 for females; 3.08 log_10_ CFU/fly, *p* < 0.001 for males; Figure 3C). These results indicate that anti-virulence strategies against tuberculosis can be effectively evaluated for efficacy in the *M. marinum–Drosophila* infection model, even though *M. tuberculosis* is not used as the pathogenic agent.

Taken together, these findings demonstrate that the yeast-drop methodology enables the detection of efficacy across diverse compound classes—including antibiotics, HDTs, and anti-virulence compounds—that are active within the physiological constraints of the *Drosophila* host and *M. marinum* infection system.

## 4 Discussion

The use of *Drosophila melanogaster* as an infection model has gained increasing attention due to its well-characterized innate immune system and the growing body of studies supporting its suitability for evaluating therapeutic efficacy (23). In drug discovery pipelines, the transition from *in vitro* screening to mammalian testing represents a critical bottleneck: many compounds active *in vitro* fail *in vivo* due to the absence of physiological complexity and host–pathogen interactions in cell-based assays (12, 24, 25). Direct testing of large compound libraries in murine models is also limited by ethical and economic constraints. In this context, *Drosophila* offers a valuable intermediate platform bridging simple *in vitro* systems and vertebrate models.

Here, we validated a new treatment methodology to screen compound efficacy against *Mycobacterium marinum* infection, the established model for active tuberculosis-like disease in *Drosophila* (22, 26). *M. marinum* shares extensive genomic and virulence homology with *M. tuberculosis*, while thriving at the fly's physiological temperature of 25 °C, unlike *M. tuberculosis*, for which infection in *Drosophila* remains unfeasible due to the incompatible temperature requirements of both species (27).

A key limitation in using *Drosophila* for TB drug screening has been the high compound cost associated with oral treatment. Conventional methods mix compounds throughout the food substrate, requiring large quantities to reach target concentrations, which substantially increases experimental costs (17, 18). To overcome this, we developed a cost-effective “yeast-drop” approach, in which a concentrated yeast paste containing the compound is deposited as a small drop in the center of a fly vial with minimal medium. This method exploits the strong natural attraction of flies to yeast-based substrates (19, 28, 29), reducing the compound used by approximately tenfold in comparison with the standard approach while ensuring ingestion.

To validate the yeast-drop methodology, we compared its performance against the standard feeding method using *M. marinum*-infected flies treated orally with linezolid and rifampicin—two well-characterized TB antibiotics—with non-treated flies as controls for each method. Results showed that both methodologies significantly improved survival and reduced bacterial burden when treated with linezolid compared with controls, confirming the reliability of the yeast-drop approach. Although the standard feeding method yielded slightly stronger effects—likely due to continuous exposure across the entire food surface—the yeast-drop technique emerged as a practical and scalable alternative, particularly when compound availability is limited. Rifampicin, however, failed to show a significant reduction in CFU counts in this particular experiment, a finding that contrasted with its performance in the subsequent benchmarking assays. We attribute this variability to its well-known light and temperature sensitivity, leading to degradation and loss of activity (30–32). Such degradation can easily occur in biological assays where flies are maintained under 12-h light/dark cycles and exposed to drug preparations over several days in complex feeding matrices such as fly media or the yeast paste.

In non-infected flies, females treated with the yeast-drop method showed reduced survival, reflecting sex-specific behavioral responses rather than toxicity. Female often exhibit stronger attraction to yeast substrates compared to males (28, 29), which, together with the dense and sticky consistency of the yeast paste during the initial hours of exposure, may increase entrapment or stress-induced mortality. Conversely, in infected flies, yeast-drop-fed female controls showed improved survival when compared with those fed standard media. This protective trend is consistent with previous findings showing immunostimulatory properties of yeast-derived β-glucans in *Drosophila* (33, 34). Together, these data validate the yeast-drop method as an efficient, low-cost, and biologically relevant oral treatment approach in the *M. marinum–Drosophila* infection model.

Sex-specific differences were also evident in treatment outcomes. Our results revealed that in treatment conditions, females showed greater efficacy of protection. In agreement with our findings, authors reported that female flies generally feed more frequently and for longer durations than males, resulting in higher compound intake (35). Moreover, dimorphisms in gut physiology and metabolism—including differences in redox state, and intestinal motility (excretion)—can alter compound absorption and clearance, helping explain sex-dependent variations in efficacy. In particular, males showed to retain defecation under stress conditions, whereas females are more prone to defecation blockage (36, 37). Such differences in intestinal motility and excretion may also affect the effective exposure time to orally administered compounds, potentially contributing to the sex-specific pharmacodynamic patterns we observed.

Using the yeast-drop method, we next evaluated benchmark compounds known to be active against *Mycobacterium tuberculosis* in the clinics, in the *M. marinum–Drosophila* infection model. Throughout this analysis, survival and bacterial burden were intentionally considered as complementary, rather than hierarchical, readouts, as they capture distinct but equally relevant aspects of compound activity, including pathogen control, host tolerance and resistance, and overall disease outcome. Compounds were categorized as antibiotics, HDTs, and anti-virulence agents. Among antibiotics, linezolid consistently produced the strongest efficacy, significantly improving survival and reducing bacterial burden in both sexes. Its favorable lipophilicity, chemical stability, and capacity to modulate immune responses likely contribute to its robust performance in our model (38, 39), which was already reported against other infections in *Drosophila* (14, 18, 40). In clinical practice, linezolid is a key component of modern multidrug-resistant and extensively drug-resistant tuberculosis regimens, though its use is limited by hematological and neurological toxicity (41). Collectively, these results support linezolid as a reliable benchmark antibiotic for validating the yeast-drop methodology, establishing a reference point for identifying compounds with comparable efficacy but improved safety profiles in higher-order models. In contrast, isoniazid and pyrazinamide were inactive, consistent with the intrinsic resistance of *M. marinum* to isoniazid, and its inability to generate pyrazinoic acid under neutral pH reached in fly hemocytes (13, 42). Not surprisingly, the standard first-line regimen to treat TB (HRZE, Rimstar^®^), which combines isoniazid (75 mg), rifampicin (150 mg), pyrazinamide (400 mg), and ethambutol (275 mg), displayed a moderate survival benefit but failed to significantly reduce bacterial burden. This reduced efficacy is likely driven by the limited activity of isoniazid and pyrazinamide in this model, together with the lower relative amount of rifampicin in the combination compared to rifampicin administered alone, rather than by an antagonistic interaction among the compounds (13, 42). Notably, ethambutol was not evaluated as a single agent in this study, which limits our ability to assess its individual contribution to the observed outcome. Moxifloxacin exhibited contrasting survival and colony-forming unit (CFU) results between sexes. In females, it induced a resistant phenotype to infection, significantly reducing bacterial burden without improving overall survival. In males, however, the drug elicited a more tolerant response, resulting in higher survival probabilities but bacterial loads comparable to the control group. Although these sex-specific differences were not observed under standard drug-testing conditions, variation in infection tolerance and resistance between male and female *Drosophila* has been extensively documented and linked to differences in immune regulation, metabolism, reproductive status, and stress responses (26, 37, 43).

HDTs represent a promising strategy in TB treatment by targeting host immune pathways rather than the pathogen itself, thereby enhancing bacterial clearance and limiting tissue damage, complementing the action of conventional antibiotics (4–6). In our model, aspirin, metformin, ibuprofen, and simvastatin all improved survival and reduced bacterial load, highlighting the conservation of host defense mechanisms between flies and mammals. Our observation that aspirin reduced CFU and improved survival in the tuberculosis model of *Drosophila* is consistent with previous findings. Low-dose aspirin reduced lung pathology and bacillary burden in the later stages of active tuberculosis in murine models, as well as improved overall survival in human tuberculosis cohorts (44, 45). Additionally, studies in Drosophila have demonstrated that aspirin can combat viral infection through activation of the STING (Stimulator of Interferon Genes) pathway, which is involved in the regulation of oxidative stress and lipid storage (46, 47). Ibuprofen treatment of TB has also been reported to reduce bacillary load and lung damage in murine models, and to extend lifespan in *Drosophila* through modulation of the Pkh2–Ypk1–Lem3–Tat2 signaling pathway, analogous to mammalian PI3K–PDK1–AKT/mTOR signaling (48–50). In our case, ibuprofen-treated flies only showed significance with respect to the control when control data were pooled, and data gained consistency. In the stratified analysis per experimental rounds, ibuprofen failed to show an effect due to increased variability. Metabolic modulators such as metformin and simvastatin also demonstrated strong activity in our model. Metformin promotes AMPK activation, autophagy, and ROS production, all of which are conserved in *Drosophila* (51–53). Simvastatin improved survival in both sexes, with significant CFU reduction in females, reflecting conserved cholesterol and lipid metabolism mechanisms between flies and mammals (54, 55). In addition, some authors have reported sex-dependent variations in lipid metabolism and its relation with stress tolerance, which may relate to the differences in CFU counts that we observed between sexes (56, 57). In contrast, doramapimod, a p38 MAPK inhibitor (58), showed no efficacy in our model. This likely reflects the essential role of p38 signaling in *Drosophila* innate immunity, where it regulates antimicrobial peptide induction and stress responses; thus, pharmacological inhibition may impair protective mechanisms rather than mitigate pathology (59–61). Additionally, doramapimod's specificity for human p38 isoforms could limit its activity against *Drosophila* homologs (p38a/p38b) (62).

To evaluate whether our model is suitable for testing anti-virulence strategies, we tested BBH7, a blocker of EsxA secretion and ESX-1 substrate export, and ethoxzolamide, an inhibitor of the PhoPR regulon. Both compounds, previously shown to reduce *M. tuberculosis* growth *in vitro* and in mice (9), improved survival and reduced bacterial load in our *M. marinum*–infected flies in both sexes. These effects likely reflect the conservation of ESX-1 secretion and the PhoPR two-component system, between *M. marinum* and *M. tuberculosis* (63, 64), and validates the model's suitability for anti-virulence compound screening.

Despite the several advantages that *Drosophila* offers as a model organism for assessing treatment efficacy, several limitations must be acknowledged. The absence of an adaptive immune system restricts *Drosophila* to modeling innate immune responses, limiting its ability to fully capture the immunopathology of human TB and narrowing the range of compounds that can be effectively tested to those targeting bacterial or innate immune components. Additionally, while the anatomical simplicity of *Drosophila* reduces physiological comparability with mammalian systems, it allows for clearer mechanistic interpretation and supports high-throughput testing, which is beneficial in early drug discovery. From a technical perspective, oral administration of compounds introduces variability in ingestion among individual flies, and lipophilic molecules may present solubility challenges. These factors can result in inconsistent exposure levels or suboptimal compound delivery. However, conducting proper dose-response experiments to identify the optimal dose and time of exposure for your specific protocol, along with supplementary qualitative methods such as colorimetric assays to confirm compound ingestion, can help validate general ingestion of the compound (65). For more precise results, advanced techniques like mass spectrometry of ingested compounds in individual flies or the Capillary Feeder Assay (CAFE) can be utilized (66, 67). Moreover, although the conservation of signaling pathway names between species suggests similarities, it does not guarantee that drug targets are conserved—especially for HDTs whose effectiveness relies on specific molecular interactions that may differ between invertebrate and mammalian orthologs. Together, these observations indicate that the *M. marinum–Drosophila* model is not intended to capture the full spectrum of anti-tuberculosis drug activity. In particular, it may not fully reflect the efficacy of compounds that depend on host-specific metabolism, adaptive immune responses, or molecular targets that are not conserved in *M. marinum*. Rather than representing a failure of predictability, this limitation defines the biological scope of the model. Within this scope, the model is well suited to identifying compounds with direct antimycobacterial activity against *M. marinum*, conserved anti-virulence mechanisms, and host-directed therapies acting on evolutionarily conserved innate immune and metabolic pathways. An additional limitation is that reductions in bacterial burden observed after treatment cannot be clearly distinguished as bactericidal or bacteriostatic effects. This distinction is particularly relevant for host-directed and anti-virulence compounds, where decreased CFU counts may result from altered infection dynamics, reduced bacterial growth, or enhanced host immunity rather than direct bacterial killing. Accordingly, this model is best suited to identifying compounds that modify disease progression, while a more detailed mechanistic classification requires follow-up studies using complementary assays. Recognizing these limitations and carefully contextualizing compound behavior within the biological and pharmacological constraints of the fly model is crucial to avoid overinterpretation of results and to ensure meaningful translational relevance.

In conclusion, the *Drosophila*–*M. marinum* infection model, combined with the yeast-drop methodology, provides a reliable, low-cost, and biologically relevant platform for the early-stage evaluation of antimycobacterial, host-directed, and anti-virulence compounds in the TB field. By capturing conserved host–pathogen interactions and enabling sex-specific analyses, this system serves as a valuable intermediate step between *in vitro* assays and mammalian models.

## Data Availability

The raw data supporting the conclusions of this article will be made available by the authors, without undue reservation.

## Supplementary material

Supplementary Figure 1 Efficacy of benchmarking compounds against TB using the yeast-drop treatment methodology in *M. marinum*-infected *D. melanogaster* model represented by experimental round. **(A)** Hazard ratios, and **(B)** bacterial load expressed as log_10_ CFU per fly in non-treated control flies (C–) and treated flies separated by true experimental rounds and not by compound category. Round 1: control flies (C–), isoniazid (INH), moxifloxacin (MFX), pyrazinamide (PZA), rifampicin (RIF), linezolid (LZD), and aspirin (ASA). Round 2: C–, doramapimod (DORA), simvastatin (SIM), metformin (MET), the PhoP inhibitor ethoxozolamide (PHOP), and the ESX-1 inhibitor BBH7. Round 3: C–, and the current standard of care (HRZE). Round 4. C–, and ibuprofen (IBU). Analyses were performed separately for males (left), and females (right). Survival data were analyzed using Cox proportional hazards regression, and CFU data using the Kruskal–Wallis test followed by Dunn's *post hoc* multiple comparisons. The technical limit of detection (LOD) for CFU quantification is 40 CFU per fly and is indicated by a gray dotted line in all CFU plots. Values below this threshold are reported as “below LOD” and, where required for log10 transformation and statistical analysis, were imputed at the LOD value. Statistical significance is indicated as follows: ns, not significant; ^*^*p* < 0.05; ^**^*p* < 0.01; ^***^*p* < 0.001.

Supplementary Figure 2 Estimated Hazard Ratios by methodology and treatment group from validation results of the “yeast-drop” treatment methodology in *M. marinum*-infected *Drosophila melanogaste*r. Estimated hazard ratios by sex within each treatment group expressed in log scale. Survival data were analyzed using Cox proportional hazards regression. Contrasts between males and females were assessed from estimated marginal means (EMMs). Standard method is shown in orange, yeast-drop method in blue. Statistical significance is indicated as follows: ns, not significant; ^*^*p* < 0.05; ^**^*p* < 0.01; ^***^*p* < 0.001.

Supplementary Figure 3 Estimated Hazard Ratios and CFU counts by sex and treatment group from validation results of the “yeast-drop” treatment methodology in *M. marinum*-infected *Drosophila melanogaste*r. **(A)** Estimated hazard ratios by sex within each treatment group expressed in log scale. Survival data were analyzed using Cox proportional hazards regression. Contrasts between males and females were assessed from Estimated Marginal Means (EMMs) **(B)** Bacterial load after treatment expressed as log_10_ CFU per fly by sex within each treatment group. Statistical comparisons were performed using the Kruskal–Wallis test followed by Dunn's *post hoc* multiple comparisons with Bonferroni correction. The technical limit of detection (LOD) for CFU quantification is 40 CFU per fly and is indicated by a gray dotted line in all CFU plots. Values below this threshold are reported as “below LOD” and, where required for log_10_ transformation and statistical analysis, were imputed at the LOD value. Females are shown in orange, males in blue. Statistical significance is indicated as follows: ns, not significant; ^*^*p* < 0.05; ^**^*p* < 0.01; ^***^*p* < 0.001.
